# Montreal’s Strategy for Hot Days: Evaluating the Effectiveness of One City’s Heat Action Plan

**DOI:** 10.1289/ehp.124-A207

**Published:** 2016-11-01

**Authors:** Nate Seltenrich

**Affiliations:** Nate Seltenrich covers science and the environment from Petaluma, CA. His work has appeared in *High Country News*, *Sierra*, *Yale Environment 360*, *Earth Island Journal*, and other regional and national publications.

Hot days can be deadly,^1^ so public health officials seek to mitigate their effects through heat action plans. These plans have been widely adopted, but little is known about how effective they really are at reducing the public health burden of high temperatures. A new study quantifies the effectiveness of one city’s plan for handling heat.^2^

**Figure:**
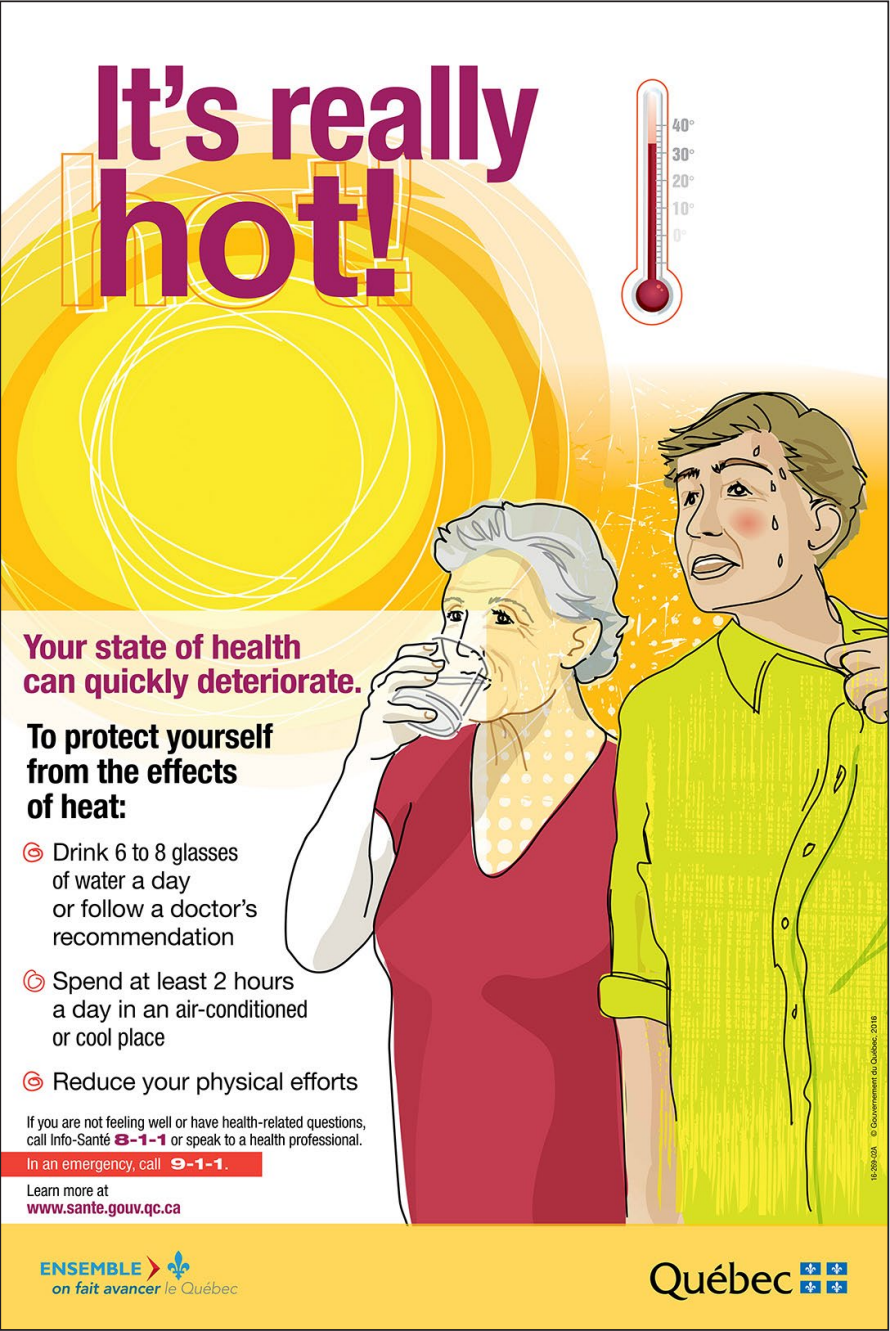
Montreal’s Public Health Department distributes educational materials as part of its heat action plan. © Gouvernement du Québec

“There are hundreds of heat action plans being implemented around the world,” says University of Washington professor Kristie L. Ebi, who did not participate in the study. “Very few are designed in a way that makes it easy to evaluate which components are associated with decreased morbidity and mortality, which makes it challenging to establish their effectiveness.”

Heat action plans are based on predetermined temperature thresholds that trigger a range of measures, from public advisories at the lowest level to opening air-conditioned shelters when temperatures are higher.^3^ In Montreal, Canada, the Public Health Department developed a heat action plan after a catastrophic 2003 heat wave in Europe killed 15,000 people in one week.^4^ Montreal’s plan kicks in when temperatures exceeding 30°C (86°F) are forecast.^5^

To assess the Montreal program’s effectiveness at reducing mortality, lead author Tarik Benmarhnia, a postdoctoral researcher at McGill University, borrowed the so-called difference-in-differences statistical framework from the field of economics. He and his colleagues compared average daily deaths on hot days before and after the plan was implemented (2000–2003 versus 2004–2007). Then they compared that value to the difference between average daily deaths on non-hot days before and after the plan was implemented.

Based on these values, the authors estimated that Montreal’s plan prevented an average of 2–3 deaths on hot days, representing more than half the deaths attributed primarily to heat by hospital coding. When they excluded deaths among people who likely would have died in subsequent weeks even without the heat—a phenomenon called mortality displacement—mortality was reduced by an average of just under 2 deaths per day.^2^

Extreme heat is known to have highly variable effects across subsets of a population.^6^ ^,^ ^7^ ^,^ ^8^ For instance, low-income homes may not have access to air conditioning, and elderly people may be socially isolated and unable to seek help if it gets too hot.^6^ The researchers therefore compared reductions achieved among men versus women, those living in low- versus high-income neighborhoods, and those above versus below the age of 65. They found that residents of low-income neighborhoods and the elderly appeared to benefit the most from the heat action plan.^2^

“Most of the benefits of this program are accumulating in populations with many of these vulnerability characteristics,” Benmarhnia says. “The benefits for people younger than 65 are very, very minor.” Future studies could seek to assess how heat action plans help other vulnerable populations such as the homeless and the mentally ill, he adds.

One important question the study does not answer is which specific components of Montreal’s program may have been responsible for any decline in deaths, notes Melanie Boeckmann, a research fellow at Düsseldorf’s Heinrich Heine University who led a 2014 review of heat plan effectiveness.^9^ And, she adds, while the difference-in-differences approach controls for potential confounders better than standard before-and-after or city-to-city comparisons, it—like any other epidemiological study—cannot prove causation.

All the same, senior author and McGill professor Jay Kaufman considers the findings persuasive. “It’s very hard to come up with an alternative explanation of what this is, if it’s not an effect of the heat action plan,” he says. “So I think this is good evidence, and more studies using this kind of design will make for even stronger evidence.”
